# Vitrification technique for female germinative tissue cryopreservation and banking

**DOI:** 10.5935/1518-0557.20190069

**Published:** 2020

**Authors:** Carlos Gilberto Almodin, Moacir Rafael Radaelli, Paula Motta Almodin, Vânia Cibelle Mingetti-Câmara, Carla Graziele da Silva

**Affiliations:** 1 Materbaby - Reprodução Humana e Genética. Maringá, Brazil; 2 Departamento de Urologia, Escola de Medicina, Faculdade Ingá, Maringá, Brazil; 3 Departamento de Análise Farmacêutica, Universidade Estadual de Maringá - UEM, Maringá, Brazil

**Keywords:** germinative tissue, female infertility, cryopreservation, vitrification

## Abstract

**Objective:**

To report on a device designed for the vitrification of germinative tissue, and a systematic vitrification/warming protocol.

**Methods:**

We obtained six fragments of cortical germinative tissue from a human ovary. We randomly chose two fragments and sent them to histological analysis. We vitrified four test samples and stored them for one week in liquid nitrogen (LN), and warmed one week later. We sent the vitrified/warmed fragments to the pathology laboratory, where they analyzed them morphologically under an optical microscope (10-40X). They analyzed the nuclear and cytoplasmic characteristics of the follicular cells, luteal layer, and stroma. The primordial and primary follicles in the fresh and vitrified/warmed fragments were counted and compared with the Mann-Whitney test (*p*<0.05).

**Results:**

There were ovarian follicles in different phases of maturation in both fresh and vitrified/warmed fragments, with a predominance of healthy-looking primordial and primary follicles. In the test fragments, the fusocellular architecture supporting the stromal cells exhibited some foci of edema, and were associated with cells with hydropic degeneration, with cytoplasmic fragmentation and eosinophilia. However, there were no signs of tissue necrosis or autolysis. There was no statistically significant difference between the number of follicles found in the control and test tissue fragments (*p*>0.05).

**Conclusions:**

There were no significant morphological changes between fresh and vitrified/warmed germinative tissue. The vitrification device and protocol tested were effective in the preservation of human follicles, and should be considered for the banking germinative tissue for the restoration of fertility of women who are submitted to life-saving sterilizing treatments.

## INTRODUCTION

Studies on the cryopreservation of germinative tissue emerged from the desire to preserve the fertility of young female patients diagnosed with aggressive diseases that needed to be submitted to life-saving, but sterilizing treatments. Since the beginning of the 20^th^ century, several authors have been studying the behavior of transplanted female germinative tissue (Gardner & Li, 1947; Lipschutz & Ponce de Leon, 1946). Animal studies demonstrated good results with transplants performed with fresh germinative tissue (Biskind & Biskind, 1948; Biskind & Kordan, 1949; Fels & Foglia, 1960). However, in order to assist patients, the germinative tissue would have to be preserved for long periods until after patient recovery, which was not possible with the techniques available at the time.

The main limitation to preserving fertility was the difficulty to develop an adequate cryopreservation technique that would keep the same morphological and functional characteristics of the fresh germinative tissue. In the 1990s, several authors reported some success using the slow-freezing technique (Moomjy & Rosenwaks, 1998; Salle *et al*., 1998). However, the results were still uncertain and the quality of the thawed cryopreserved specimens was poor (Aubard *et al*., 1998). In 2004, Almodin and his group were the first to report on the successful cryopreservation and thawing of fragments of germinative tissue with the slow-freezing technique, which were subsequently orthotopically transplanted into an ovary that had been kept in place and submitted to sterilizing levels of radiation. Studies carried out with two different animals resulted in the live birth of naturally conceived sheep (Almodin *et al.*, 2004a) and rabbits (Almodin *et al.*, 2004b) after transplantation of cryopreserved germinative tissue into the atrophic irradiated ovary. Since then, the cryopreservation and grafting of germative tissue has been carried out all over the world, with several live births being recorded (Donnez *et al*., 2004; Meirow *et al*., 2005; Demeestere *et al*., 2007; Andersen *et al*., 2008; Donnez & Dolmans, 2015). However, shortly after birth, ovarian function came to a halt (Donnez *et al*., 2004). However, it is unclear, if the slow-freezing technique or ischemia time of the transplant technique compromised the number of oocytes available (Baird *et al*., 1999).

Vitrification is an ultra rapid freezing technique that makes use of minimum amounts of cryoprotectants, which has emerged to revolutionize the cryopreservation process, with excellent and predictable results in embryos (Kuwayama *et al*., 1992). Vitrification is increasingly becoming the equipment of choice for the cryopreservation of gametes in many human reproduction centers around the world. Among the advantages of vitrification is the time required with the conventional slow-freezing procedure. A recent study showed that an average of three to four hours was required to cryopreserve male germinative tissue, versus just 30 min for vitrification (Radaelli *et al*., 2017). Slow-freezing equipment is also highly costly and complex, while the vitrification equipment is relatively cheap and the procedure can be learned much faster. The vitrification equipment is also light and can be easily transported, so that tissue freezing can be conducted wherever the patient is, even if the site is far from the banking facility (Baert *et al*., 2012).

The successful vitrification of embryos (Almodin *et al*., 2010), oocytes (Almodin *et al*., 2015), and male germinative tissue (Radaelli *et al*., 2017) using the Vitroequip method have already been reported by our research team. Radaelli *et al*. (2017), found that the morphology of prepuberal male germinative tissue in rats was adequately cryopreserved with both slow freezing and vitrification. Nonetheless, recent efforts have focused on minimizing the loss of oocytes from freezing damage, with adequate procedures involving minimal use of cryoprotectants. Previously, Kagawa *et al*. (2009) reported on a method designed for the cryopreservation of ovarian tissue (Cryotissue), which yielded high oocyte survival in human tissue, indicating the vitrification method's potential for clinical use.

Based on these previous experiences, it has become clear that proper devices and protocols for the cryopreservation of germinative tissue are required to ensure the safety and reproducibility of the process (Kagawa *et al*., 2009; Radaelli *et al*., 2017). Therefore, the objective of this study is to report on the Vitroequip method for the vitrification of male and female germinative tissue, which provides easy tissue handling, simplifying its exposure to liquid nitrogen, and optimizing storage space. Moreover, a systematic description of the vitrification protocol detailing all the stages of the process is also presented.

## MATERIALS AND METHODS

We performed all the vitrification procedures at the human reproduction laboratory of Materbaby - Reprodução Humana, Maringá, Brazil. Germinative tissue samples were obtained from a human ovary of a female patient, aged 32 years, who underwent total hysterectomy surgery due to a benign uterine disease, with complete ovaries removal. The patient had no interest in cryopreserving her genetic material, and donated her ovaries to research after signing an informed consent form.

### Germinative tissue preparation

**Figure 1:**
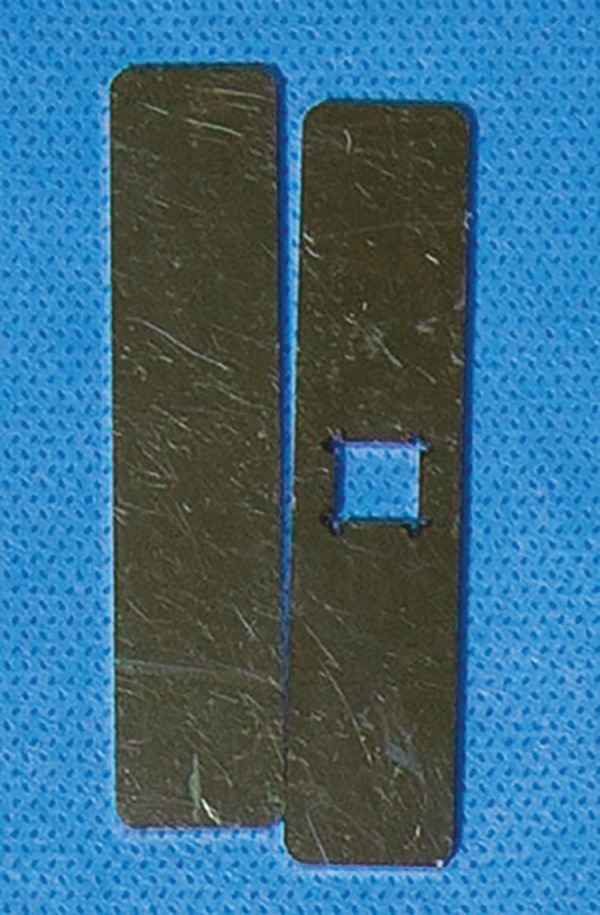
Device for the dissection of ovarian tissue.

**Figure 2:**
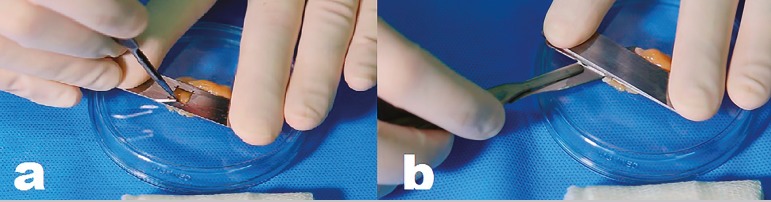
a) Incision of the germinative tissue with a scalpel within the dissection area; b) Sectioning of the germinative tissue to separate the top 1 mm thick layer containing the ovarian cortex.

We obtained ovary tissue standardized samples using a specially developed dissection device, made up by two stainless steel plates measuring 6.5 cm x 1.4 cm x 1 mm thick. One of the plates has a central opening measuring 0.7 cm x 0.7 cm (Fig. 1). The ovarian tissue was placed on a Petri dish, which was them covered with the open metal plate, exposing the tissue`s central area. We then cut the tissue along the borders of the opening with an 11-blade (Fig. 2a). After sectioning opening`s periphery, the second stainless steel plate was placed over the first plate to enable dissection of the tissue`s top layer, just removing a 1 mm thick fragment of the ovarian cortex containing the germinative tissue (Fig. 2b). We obtained a total of 6 fragments; two of which were randomly chosen, individually stored in flasks containing 10% buffered formalin solution, and sent directly to the pathology laboratory to be processed with hematoxylin-eosin and histology analysis (control specimens), while the other four fragments were separated for vitrification (test specimens).

### Vitrification equipment

**Figure 3:**
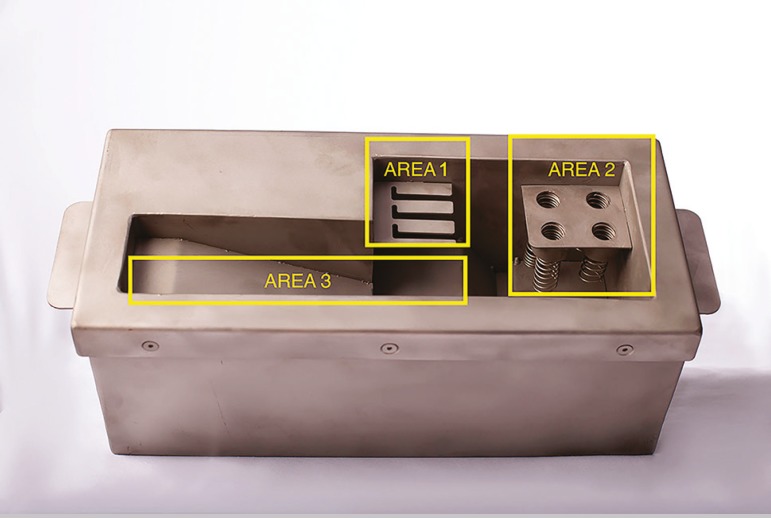
Vitrification equipment. Area 1: vitrification of oocytes and embryos; Area 2) lodging of cryotubes for the vitrification of germinative tissue; and Area 3) vitrification ramp.

**Figure 4:**
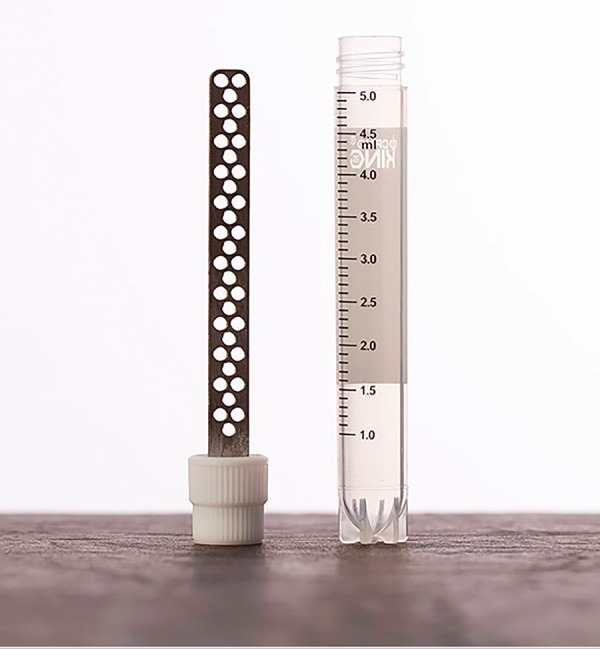
Stainless steel stem for the vitrification of germinative tissue.

The vitrification equipment used in this study was the Vitroequip (Ingamed, Maringá, Brazil). This equipment was originally developed for the vitrification of embryos and oocytes, which recently received modifications to enable germinative tissue vitrification. It has a medical stainless steel box, which holds 0.8L of liquid nitrogen (LN). The box has 3 working areas: 1) vitrification of oocytes and embryos; 2) lodging the cryotubes for the vitrification of germinative tissue; and 3) supporting ramp, where the material to be vitrified is handled, and the storage rod is placed before being transferred to an LN tank (Fig. 3). Moreover, stainless steel vitrification stem has openings along its surface, embedded in a cryovial plastic lid, specially designed for the placement of germinative tissue, to facilitate vitrification and storage (Fig. 4).

### Vitrification procedure

**Figure 5:**
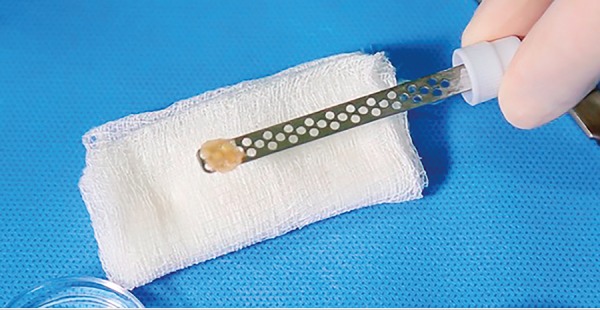
Germinative tissue fragment placed on the vitrification stem ready for vitrification.

We placed the four test ovarian cortex fragments individually into cryovials, containing the equilibrium solution V-I (Ingamed, Maringá, Brazil), made up of 7.5% ethylene glycol and 7.5% dimethyl sulfoxide (DMSO), for 10 min at 4°C. Then, we removed the fragments from the solution with dissection tweezers, dried on a sterile dressing to remove excess cryoprotectant, and immediately immersed into the V-II vitrification solution (Ingamed, Maringá, Brazil), made up of 15% ethylene glycol, 15% DMSO and sucrose 0.5 M for 2 min at 4°C. We then dried the ovarian tissue fragment on a sterile dressing and carefully placed onto the surface of the vitrification stem, with the tunica albuginea in contact with the stem (Fig. 5). We them carefully immersed the stem with the fragments into LN in area 3 of the vitrification device, between 10 and 15 sec until the fragments were completely vitrified. After that, we placed the stems inside cryotubes filled with LN, lodged in area 2. The tube was tightly closed, removed, and immediately plugged with the storage rod inside LN in area 3, preventing major changes in temperature. Later, we transferred the rod to a LN storage tank.

### Warming procedure

**Figure 6:**
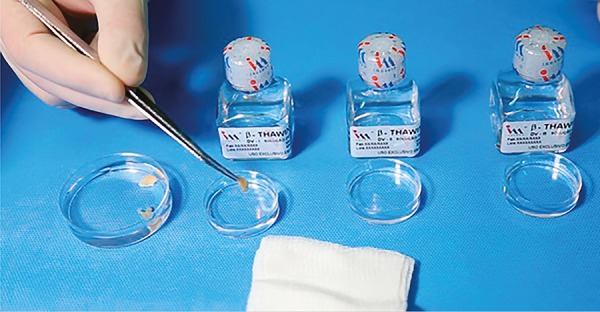
Germinative tissue fragments being sequentially immersed into the warming solutions and dried during the warming process.

After one-week storage in LN, the vitrified tissue fragments were warmed. The stems were removed from the cryovials and immediately placed in 15 ml conical centrifuge tubes, containing the DV-I warming solution (Ingamed, Maringá, Brazil) at a temperature of 37°C for 5 min. After that, the ovarian tissue fragments were removed from the solution with dissection tweezers, and sequentially placed into Petri dishes containing the warming solutions DV-II for 5 min, DV-III for 5 min, again in DV-III for 6 min, and finally in DV-III for another 5 min. After each immersion, the fragments were dried on a sterile dressing before being immersed into the next solution (Fig. 6). The dried warmed tissue fragments were individually stored in flasks containing 10% buffered formalin solution and immediately sent to the pathology laboratory to be processed for histological analysis.

### Histological analysis

Each fragment was dehydrated with ethanol, and embedded in paraffin. Each slide containing a fragment was prepared, stained with hematoxylin-eosin, and analyzed under an optical microscope (Olympus BX Series), with magnification between 10 to 40X. Nuclear and cytoplasmic characteristics of the follicular cells, the luteal layer, and the stroma were analyzed. Tissue preservation was observed in relation to: a) initial alterations of hydropic degeneration, identified by cytoplasmic swelling and edema of the supporting connective tissue; b) signs of severe tissue damage, determined by cytoplasmic and nuclear fragmentation; c) definitive signs of irreversible tissue damage, characterized by disappearance of cell and nucleus contours; and d) counting of primordial and primary follicles morphologically considered normal.

### Statistical analysis

The data on the number of follicles in the fresh and vitrified/warmed germinative tissue fragments were statistically analyzed, with the aid of the Statistica Single User software, version 13.2. Means and standard deviations were calculated, and the results were compared with the Mann-Whitney test for non-parametric distributions, at a level of significance set at 5% (*p*<0.05).

## RESULTS

Table 1 presents the number of primordial and primary follicles found in the fresh (control) and vitrified-warmed (test) fragments of germinative tissue. There were no statistically significant difference between the fresh and the vitrified/warmed fragments (Table 2).

**Table 1:** Number of follicles found in the fragments of fresh and vitrified-warmed ovarian cortex

	Total follicles	Primordial follicles	Primary follicles
**Control fragment 1**	10	9	1
**Control fragment 2**	26	22	3
**Test fragment 1**	35	28	7
**Test fragment 2**	29	23	6
**Test fragment 3**	16	12	4
**Test fragment 4**	70	60	10

Follicle counting in 10 fields (magnification 10X).

**Table 2:** Statistical comparison of the number of follicles found in the control and test fragments of germinative tissue

Variables	Test fragments (n=4)	Control fragments (n=2)	p*
	Mean±SD	Mean±SD	
**Total follicles**	37.50±23.07	18.00±11.31	0.2472
**Primordial follicles**	30.75±20.61	15.50±9.19	0.2472
**Primary follicles**	6.75±2.50	2.00±2.83	0.1649

* Mann-Whitney test not significant considering level of significance of 5%.

### Fresh ovarian tissue

**Figure 7:**
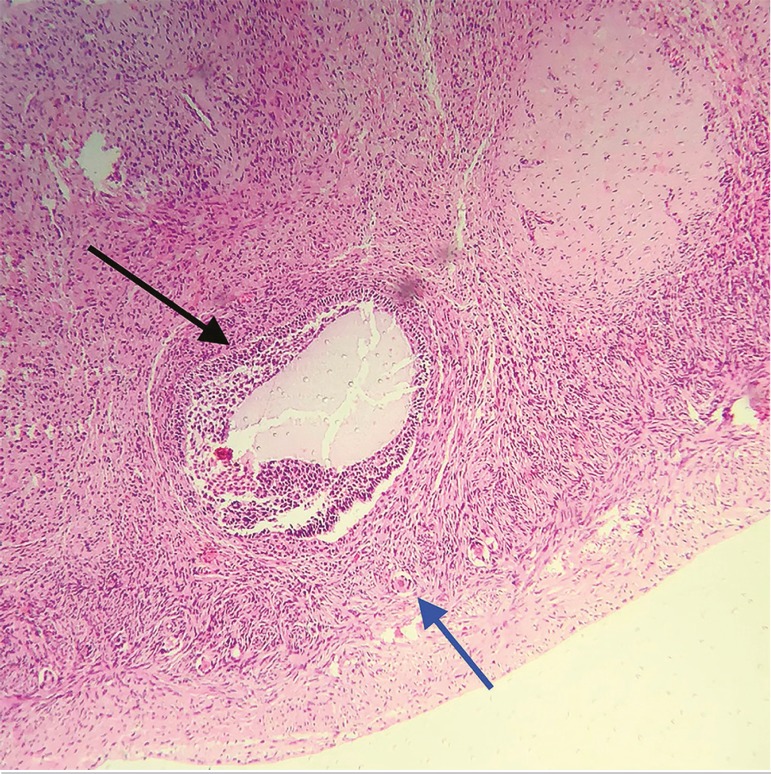
Fresh germinative tissue fragment histology slide (control), showing a primordial follicle (black arrow) and primary follicle (blue arrow). Magnification 10X.

The macroscopic assessment of the fresh ovarian tissue fragments revealed pink‐white elastic tissues with fine granular surfaces. Microscopically, the tissue presented preserved morphology in all the fields examined, with a well-defined germinative epithelium, albuginea and cortical tissue granule‐albican bodies. There were also ovarian follicles in different phases of maturation, with a predominance of healthy-looking primordial and primary follicles. The fusocellular architecture supporting the stromal cells was trophic, and there were no signs of hydropic degeneration, edema, and necrosis or tissue autolysis (Fig. 7).

### Vitrified ovarian tissue

**Figure 8:**
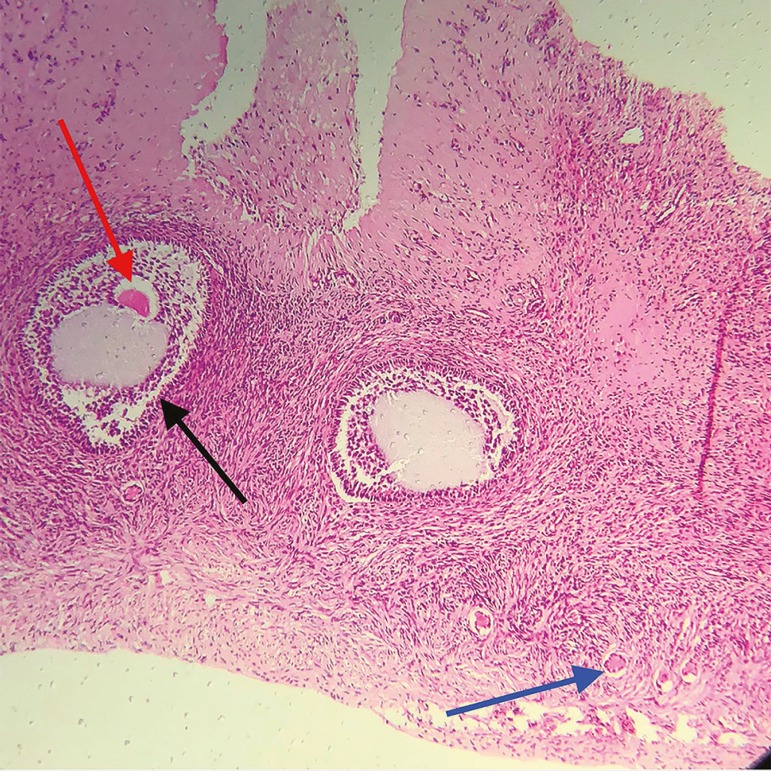
Histological slide of a vitrified/warmed germinative tissue fragment (test), showing a primordial follicle (black arrow), primary follicle (blue arrow), and a fully developed oocyte (red arrow). Magnification 10X

The macroscopic assessment of the test fragments revealed white, elastic tissues with fine granular surfaces. Microscopically, the fragments presented preserved global architecture. The cortex was organized, and there were ovarian follicles in different phases of maturation, with a predominance of healthy looking primordial and primary follicles. The fusocelular architecture supporting the stromal cells exhibited some foci of edema, and was associated with cells with hydropic degeneration, with cytoplasmic fragmentation and eosinophilia. However, there were no signs of tissue necrosis or autolysis. In test fragment 4, there was a small focus of tissue necrosis, with loss of cellular and nuclear contours in the periphery of the tissue (Fig. 8).

## DISCUSSION

For the past 15 years, scientists around the world have been discussing the feasibility of preserving ovarian tissue and creating germinative-tissue banks, with the intention of recovering the fertility of young people submitted to life-saving but sterilizing treatments (Bedaiwy & Falcone, 2004). Differently from previously believed, we know now that we can obtain best results when only the ovarian cortex of the germinative tissue is cryopreserved, rather than the whole ovary (Sugimoto *et al*., 2000; Bedaiwy & Falcone, 2010).

An important question about the transplant of cortical strips concerns finding a suitable place to host the tissue after freezing, and how successful the procedure would be. Initially, both ovaries were removed before treatment began and cryopreserved, with several authors attempting heterotopic germinative tissue transplantation for the future development of follicles and the harvest of oocytes (Oktay *et al*., 2004). Heterotopic implants, either in the patient herself, e.g., in the subcutaneous area of the forearm (Oktay *et al*., 2001), or in immunocompromised mice (Oktay *et al*., 2000), have been previously attempted. However, the procedure involved safety risks and was too difficult to reproduce to justify the creation of germinative tissue banks.

Almodin *et al.* (2004 a,b) demonstrated that the removal of only one of the ovaries prior to chemotherapy or radiotherapy could be sufficient for the harvest of ovarian cortex fragments for cryopreservation. By keeping the contralateral ovary in place, it could be later used as the natural recipient for the cryopreserved tissue after the patient recovers from her treatment. By transplanting the cryopreserved ovarian cortex fragments into a depleted, previously irradiated ovary, the authors demonstrated that natural fertility could be restored, both in sheep (Almodin *et al*., 2004a) and in rabbits (Almodin *et al*., 2004b), opening the doors for the creation of germinative tissue banks. Nowadays, the implantation technique, as well as the viability of the implanted tissue is already well documented (Silber *et al*., 2010; 2015; Silber, 2012; 2016).

Vitrification is a rapidly developing technology that is increasingly being used for the cryopreservation of gametes, embryos (particularly blastocysts) and germinative tissue. Vitrification has already been demonstrated to be a reliable strategy for the cryopreservation of embryos (Almodin *et al*., 2010) and oocytes (Cobo *et al*., 2008; Cobo *et al*., 2010; Almodin *et al*., 2015). Since the development and consolidation of vitrification as an alternative to slow-freezing technique, efforts have now been focused on the development of vitrification devices and supplies that can enable the cryopreservation of germinative tissue, with results comparable to those already reported for oocytes and embryos. In a recent paper by our group, prepuberal male germinative tissue was successfully cryopreserved using the same cryoprotectants described in the present study. However, one of the technical difficulties met in the execution of the vitrification protocol was the handling of testicular tissue - due to the lack of a specific vitrification device; the tissue was simply plunged into LN (Radaelli *et al*., 2017).

Based on a previous report (Kagawa *et al*., 2009), the vitrification devices used in the present study were developed to offer a complete solution to germinative tissue vitrification. The cutting device was designed to prepare the ovarian tissue with a standard size and thickness, ensuring that only the ovarian cortex, where the follicles are located, is harvested, improving the effectiveness of the vitrification process (Kagawa *et al*., 2009). The new vitrification stem enables ovarian cortex, which has a very soft consistency, to be easily placed and remain flat during its introduction into virgin LN, resulting in optimal tissue-LN contact. Since the cryovials, where the vitrification stem are placed, are themselves immersed in LN inside the equipment and filled with LN, the whole process can be carried out without temperature loss, sufficing to close the lid and plug the cryovials in the storage rod and transfer them to an LN tank.

The device also enables tissue warming, since the stem can be easily removed from the cryovials, safely and easily placed into another cryovial containing the initial warming solution. One important aspect of the protocol described here concerns the drying process of tissue fragments, along with each vitrification/warming step. As described before (Radaelli *et al*., 2017), the constant drying process between the different tissue immersion into the solutions can enhance tissue preservation, and later benefit tissue grafting after warming.

The female germinative tissue vitrification-warming protocol described here demonstrated that the warmed tissues suffered only minor damage, without compromising tissue morphology, keeping its architecture practically intact, with high follicle survival rates. In test fragment #4, the small focus of tissue necrosis, with loss of cellular and nuclear contours found in the tissue's periphery, might be the result of overexposure to LN during vitrification. Nonetheless, the tissue was morphologically sound with a large number of primordial and primary follicles (Table 1). In addition, fully developed oocytes were also observed in the vitrified/warmed tissue fragments (Fig. 8). These excellent results, corroborate those results found in a previous report (Kagawa *et al.*, 2009), and support the use of the equipment, solutions, and the different steps used along the vitrification process described here. Future studies by our group should now focus on the grafting of vitrified/warmed germinative tissue, to ascertain tissue functionality.

## CONCLUSIONS

Based on our results, the vitrification devices, and the protocol described here were effective in the cryopreservation of follicles located on the cortex of the human ovary, and should be considered for the banking of germinative tissue, used to restore the fertility of young women who are submitted to life-saving, but sterilizing treatments.

### Support

The vitrification devices and media used along this trial were kindly supplied by Ingámed Materiais Médico-Hospitalares LTDA (Maringá, Brazil).
